# Brain barriers and functional interfaces with sequential appearance of ABC efflux transporters during human development

**DOI:** 10.1038/s41598-017-11596-0

**Published:** 2017-09-14

**Authors:** Kjeld Møllgård, Katarzyna M. Dziegielewska, Camilla B. Holst, Mark D. Habgood, Norman R. Saunders

**Affiliations:** 1 Department of Cellular and Molecular Medicine, Faculty of Health and Medical Sciences, University of Copenhagen, Blegdamsvej 3, DK-2200 Copenhagen, Denmark; 2 Department of Pharmacology & Therapeutics, University of Melbourne, Parkville, Victoria, Australia

## Abstract

Adult brain is protected from entry of drugs and toxins by specific mechanisms such as ABC (ATP-binding Cassette) efflux transporters. Little is known when these appear in human brain during development. Cellular distribution of three main ABC transporters (ABCC1, ABCG2, ABCB1) was determined at blood-brain barriers and interfaces in human embryos and fetuses in first half of gestation. Antibodies against claudin-5 and -11 and antibodies to α-fetoprotein were used to describe morphological and functional aspects of brain barriers. First exchange interfaces to be established, probably at 4–5 weeks post conception, are between brain and embryonic cerebrospinal fluid (eCSF) and between outer surface of brain anlage and primary meninx. They already exclude α-fetoprotein and are immunopositive for both claudins, ABCC1 and ABCG2. ABCB1 is detectable within a week of blood vessels first penetrating into brain parenchyma (6–7 weeks post conception). ABCC1, ABCB1 and ABCG2 are present at blood-CSF barrier in all choroid plexuses from first appearance (7 weeks post conception). Outer CSF-brain interfaces are established between 9–11 weeks post conception exhibiting immunoreactivity for all three transporters. Results provide evidence for sequential establishment of brain exchange interfaces and spatial and temporal timetable for three main ABC transporters in early human brain.

## Introduction

An important feature of the developing brain is that it is separated from the general internal environment of the embryo and fetus by a complex series of interfaces composed of morphological structures, cellular transporters and various channels in cell membranes of each interface. Circumferential tight junctions sealing the intercellular space between cells of the interfaces create barriers with a diffusion restraint for lipid insoluble molecules. This diffusion restraint allows cellular transport mechanisms in the barrier to be established to provide the functional mechanisms that control the internal environment of the brain. These mechanisms are often referred to colloquially as “the blood-brain barrier”. However, this is a misleading simplification, since there are several distinct and unique interfaces protecting the brain. The two most well described barriers are the blood-brain barrier proper, with tight junctions between endothelial cells of brain vasculature and blood-CSF (cerebrospinal fluid) barrier with tight junctions between choroid plexus epithelial cells^1^. In addition to these two barriers there are another three main interfaces and a fourth that is present only in the embryonic brain (Supplementary Figure 1). The key structural component of all of these interfaces, except the CSF-brain interface, is the presence of tight junctions^2^. These barrier interfaces are described in more detail and illustrated in Supplementary Figure 1.

Compared to studies of the adult brain, the developing brain has been somewhat neglected until recent years. It is clear that many of the mechanisms present in the adult brain are not only present in the developing brain, but some may be functionally more active^1, 3^. There is also increasing evidence that some of the mechanisms may be exclusive to the developing brain leading to the concept that the brain develops within a unique environment that is appropriate for different stages of its development^1, 4^. This is a far cry from the long-standing dogmatic evidence-free belief that “the” blood-brain barrier is leaky or absent in the embryo or even in the newborn (for discussion see ref. 5).

The two largest families of transporters in the barrier interfaces of the brain are the solute linked carriers (SLCs) and the ATP-binding cassette (ABC) transporters. In the mammalian brain the former are characteristically influx transporters and the latter efflux transporters. The influx transporters have the important role of controlling the supply of nutrient molecules such as glucose, amino acids, monocarboxylates and key metal ions that are essential for the normal development and function of the brain^1^. The efflux transporters play a key role in the protective functional properties of the barrier^6, 7^ by removing metabolic products and preventing the entry of an astonishing number of potential toxins as well as most therapeutic drugs to which the brain may be exposed^8^. ABC efflux transporters are ubiquitously distributed throughout the organs and tissues of the body. Forty-nine members of the ABC protein superfamily have been described^9^. In the adult at the blood-brain barrier proper the efflux transporters that have been identified and appear to be of particular functional importance are: ABCB1 (also known as P-glycoprotein or Multidrug Resistance Protein 1, MDR1) and ABCG2 (Breast Cancer Resistance Protein, BCRP) (cf. ref. 10). ABCC2 (Multidrug Resistance Protein 2, MRP2) and ABCC4 (MRP4) have also been demonstrated at this barrier^7^. At the blood-CSF barrier ABCC1 (Multidrug Resistance Protein 1, MRP1) appears to be the predominant efflux transporter, but ABCC4 (MRP4) and ABCG2 (BCRP) have also been shown to be present^7, 11^.

Of all the numerous cellular mechanisms that are present at various brain interfaces the least studied, particularly in the human embryo and fetus, are these efflux mechanisms. Efflux mechanisms are of considerable significance, not only because they are fundamental biological mechanisms important for normal function in the adult and developing brain but also because of their clinical relevance as potential protective mechanisms in the brain(see Discussion). In this study we report on the immunohistochemically defined spatial and temporal distribution of three key ABC transporters (ABCB1, ABCC1, ABCG2) in human embryonic and fetal brain. We describe their emergence and in some cases decline in the barrier interfaces of the developing human brain from the period of development before vascularization of the brain (i.e. before the advent of the blood-brain barrier itself), through the first appearance of the choroid plexuses, the first stages of CSF secretion and differentiation of the outer meningeal interfaces with subsequent establishment of the subarachnoid space.

## Results

Adult brain is separated from the periphery by a set of morphological and physiological mechanisms that are present at various interfaces between the brain parenchyma and surrounding fluids. There are 5 main interfaces identified in the adult brain: (a) *The arachnoid barrier*, (b) *The blood-brain barrier*, (c) *The blood-CSF barrier*, (d) *Circumventricular organs (CVO barrier)*, (e) *The adult inner CSF-brain interface*. An additional sixth interface, (f) *The embryonic CSF-brain interface*, is present only in the fetus. A brief summary is provided in the Supplementary Material (Supplementary Figure 1) in order to facilitate the understanding of results.

Here we present the data describing the sequential appearance of these barriers in the brain from the earliest embryonic stage (4–5 weeks post conception, wpc) to mid-gestation. The results are based on the individual barriers and interfaces as they first become established. We used immunopositivity for the fetal protein, α-fetoprotein (AFP) to indicate the diffusional restraint to a large hydrophilic molecule (marker of passive permeability) and the distribution of ABC efflux transporters as a marker of potential functionality of individual interfaces.

### The earliest brain interfaces (5 wpc): Primary meninx, eCSF-brain interfaces and blood-brain barrier proper

**Figure 1:**
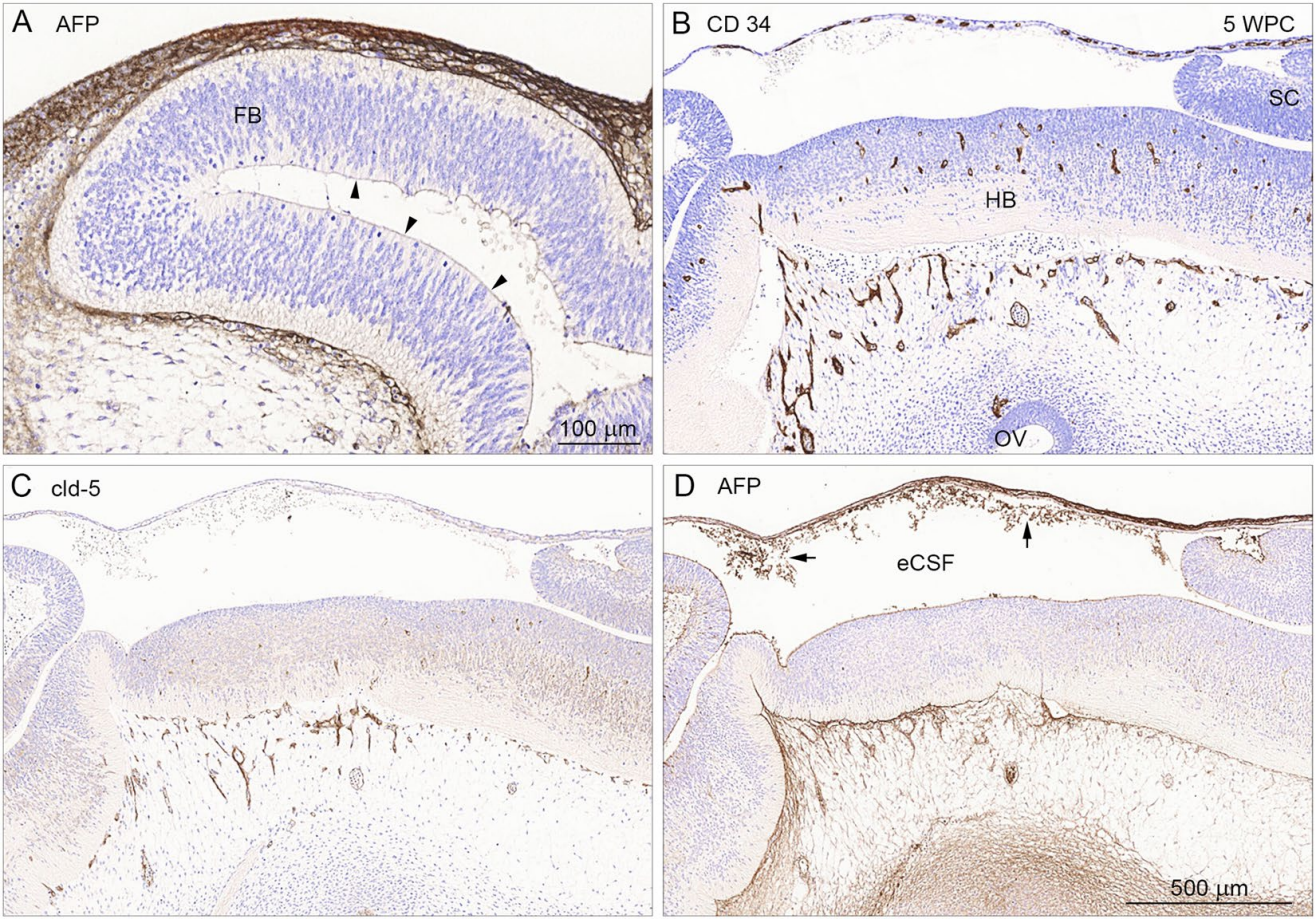
Markers for a large hydrophilic molecule (AFP), blood vessels (CD 34) and claudin-5 positive tight junctions (cld-5) in consecutive sagittal sections of a 5 wpc human embryonic brain anlage. Immunostaining for AFP (in **A)** in a sagittal section of 5 wpc human forebrain (FB) shows strong AFP signal in the loose mesenchyme surrounding the outer surface of the brain anlage and a weaker immunopositivity on the surface of the inner eCSF-brain interface (arrowheads). Note that the brain parenchyma in the forebrain is devoid of AFP immunostaining. In the midbrain and hindbrain (HB) blood vessels have already begun to penetrate the brain parenchyma as illustrated by CD 34 positive immunostaining (**B**), and display tight junctions as shown by claudin-5 positive immunostaining (**C**). The integrity of the early blood-brain barrier proper is further supported by lack of any perivascular AFP immunostaining in mid- and hindbrain (**D**). Note precipitated AFP (arrows) in the eCSF indicating its presence there. Abbreviations: eCSF: embryonic CSF; FB: forebrain; HB: hindbrain; OV: otic vesicle; SC: spinal cord, WPC: weeks post conception. (**A**–**D**) Same magnification. *Scale bar*: 500 μm.

By 5 wpc the neural tube has closed around a fluid-filled neuronal cavity, initially filled with amniotic fluid but progressively modified and referred to as eCSF (embryonic cerebrospinal fluid)^12^ before the appearance of the choroid plexuses but also at a time when the brain tissue has barely begun to be vascularized. The first exchange interfaces that are present in and around the developing brain are: (i) between the brain and the trapped fluid (eCSF) resulting from neurotube closure that in humans occurs around 26 days post conception (dpc) and (ii) between the brain and vascular, neural crest-derived loose mesenchyme forming the primary meninx at the outer surface of the brain anlage^13^. At this stage of development the forebrain is still devoid of vasculature (Fig. 1).

One of the first fetal plasma proteins that is present in the human embryonic circulation is the fetal protein α-fetoprotein (AFP)^14, 15^. A sagittal section through 5 wpc human embryonic forebrain, stained with antibodies to AFP is shown in Fig. 1A. As can be seen a strong immunopositive signal is detected in the primary meninx on the outside of the forebrain anlage. There is also a fainter immunostaining on the surface of the inner eCSF-brain interface (Fig. 1A). Brain parenchyma is devoid of AFP staining indicating that embryonic forebrain, even at such an early stage of development, is already separated from entry of plasma proteins from the periphery. However, at the same stage of development (5 wpc) in the hind- and midbrains the first blood vessels have penetrated into the brain parenchyma. Antibodies to CD 34 demonstrate the presence of blood vessels (Fig. 1B) while antibodies to claudin-5 demonstrate tight junctions present in these blood vessels (Fig. 1C). The tightness of these early penetrating blood vessels was further confirmed by staining consecutive sections with antibodies to AFP (Fig. 1D). This demonstrated that AFP was also kept out of the brain parenchyma in both mid- and hindbrains. This “tightness” of the blood-brain barrier, as demonstrated by claudin-5 positive endothelial cell tight junctions and exclusion of AFP from perivascular areas, persisted throughout the period investigated (Fig. 4A and ref. 16 based on the same material).

### ABC transporters

Antibodies to three main ABC transporters: ABCB1, ABCC1, ABCG2 were used to identify their distribution at these very early stages of human embryonic development. ABCB1 was not present in any parts of the brain investigated, but was present in the placenta. Immunoreactivity for ABCB1 appears in blood vessels about one week later than for the other two transporters (see below), and all three transporters show consistent positive signals on blood-brain barrier at least until 13 wpc.

**Figure 2:**
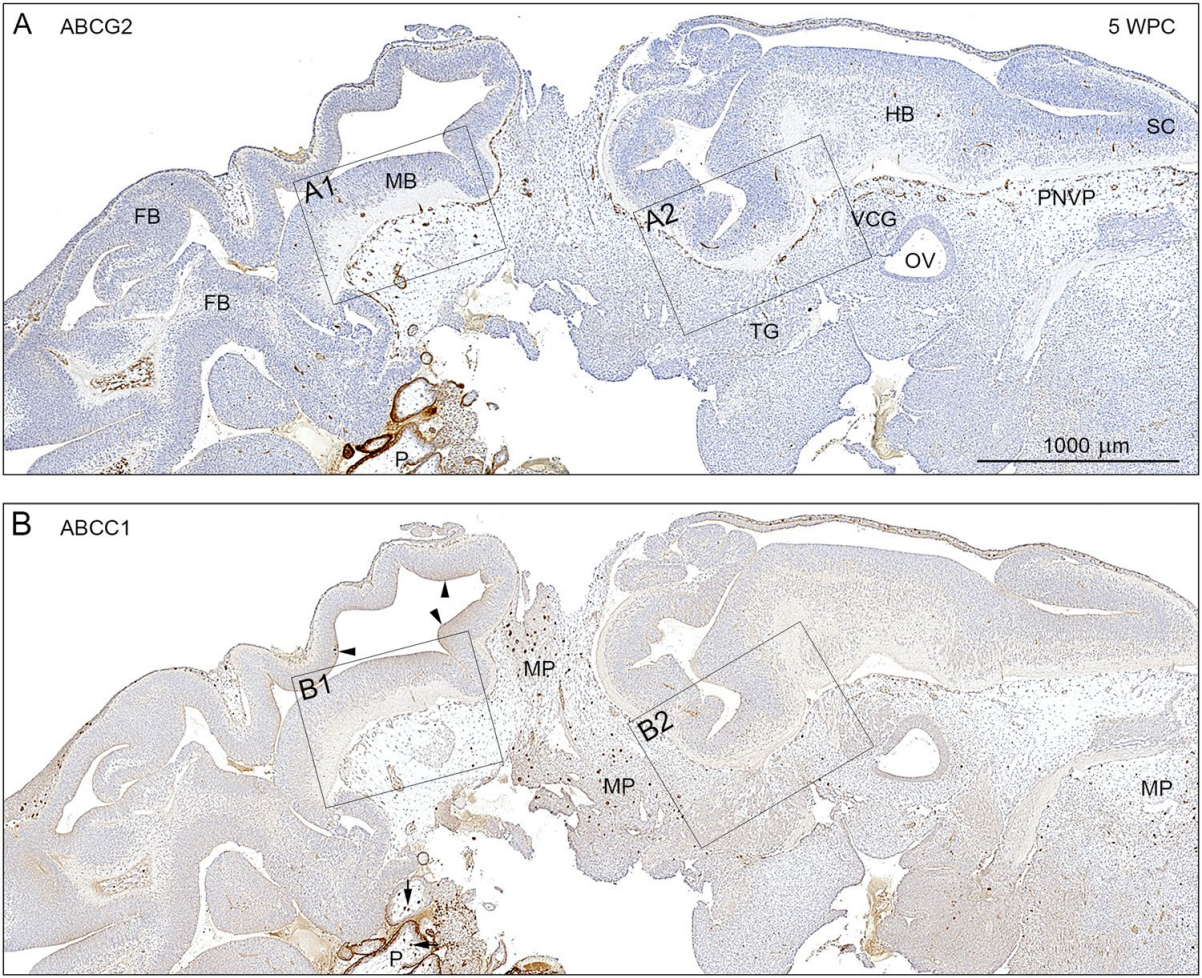
Distribution of ABCG2 and ABCC1 immunoreactivity in consecutive sagittal sections of a 5 wpc human embryonic brain anlage. Immunostaining for ABCG2 (**A**) and ABCC1 (**B**) shows a dense perineural vascular plexus (PNVP) in the ventral part of the upper spinal cord (SC), the hindbrain (HB) and the midbrain (MB) but not in the dorsal forebrain, which lacks vascularization at this time. Note the many large tissue macrophages (MP) stained for ABCC1 in the loose vascular mesenchyme surrounding the brain anlage. The eCSF-brain interface is also immunopositive for ABCC1 (arrowheads in B). The boxed areas (A1, A2, B1, B2) are shown in higher magnification in Fig. 3. The specificity of the antibodies is demonstrated in placental tissue (P) included in the sections. The syncytiotrophoblastic cell layer is strongly immunopositive for ABCG2 and ABCC1, and the prominent placental stromal macrophages (Hofbauer cells) are immunopositive for ABCC1 (arrows). Abbreviations: FB: forebrain; MB: midbrain; HB: hindbrain; MP: macrophages; OV: otic vesicle; P: placental tissue; PNVP: perineural vascular plexus; SC: spinal cord; TG: trigeminal ganglion; VCG: vestibulo-cochlear ganglion, WPC: weeks post conception. (**A**,**B**) Same magnification. *Scale bar*: 1000 μm.

**Figure 3:**
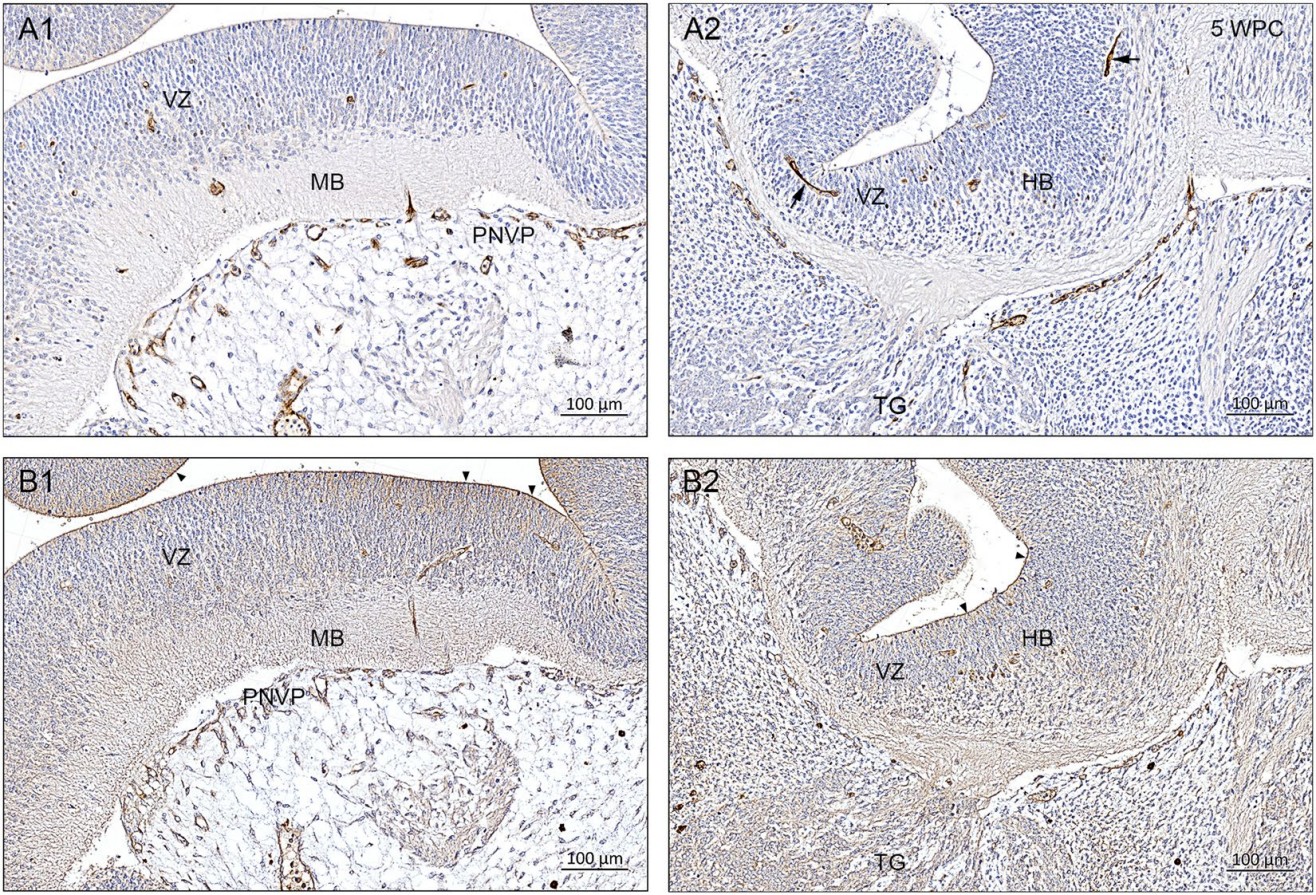
Distribution of ABCG2 and ABCC1 immunoreactivity in a 5 wpc human embryonic brain anlage at high magnification. The boxed areas (A1, A2, B1, B2) from Fig. 2 are shown in higher magnification. Blood vessels showing ABCG2 and ABCC1 immunopositive endothelial cells from the perineural vascular plexus (PVNP) sprout to form radial branches that penetrate the marginal zone and then after reaching the ventricular zone (VZ) elongate as tangentially oriented longitudinal branches. Two such branches are seen in A2 (arrows). The eCSF-brain interface is outlined by ABCC1 immunoreactivity (arrowheads in B1 and B2). Abbreviations: HB: hindbrain; MB: midbrain; PNVP: perineural vascular plexus; TG: trigeminal ganglion; VZ: ventricular zone. WPC: weeks post conception. *Scale bar*: 100 μm in all panels.

Immunostaining for ABCG2 (Fig. 2A) shows positive signal in a dense perineural vascular plexus in the ventral part of the upper spinal cord, the hindbrain (Fig. 3A2/B2) and the midbrain (Fig. 3A1/B1) whereas the dorsal forebrain is not yet vascularized as described above. Placenta is also very strongly stained for both transporters (Fig. 2A,B). Distribution of the immunostaining for ABCC1 (Figs 2B and 3B1,B2) is similar although it appears to be weaker than that for ABCG2. However tissue macrophages, present in primary meninx are especially strongly positive for ABCC1 (Fig. 2B). The eCSF-brain interface is also stained for ABCC1 (arrowheads in Fig. 2B). At this interface immunostaining for ABCG2 is less pronounced and not so uniform (Fig. 2A). At higher magnification, shown in Fig. 3, both transporters are clearly visible in all blood vessels in both the perineural vascular plexus and brain tissue.

The specificity of the antibodies was demonstrated in placental tissue (P) included in the sections (Fig. 2A,B). The syncytiotrophoblastic cell layer is strongly positive for both ABCG2 and ABCC1, and the prominent placental stromal macrophages (Hofbauer cells) are positive for ABCC1 (arrows in Fig. 2B). ABCC1 staining and lack of immunoreactivity for ABCG2 of both tissue macrophages and Hofbauer cells are clearly shown in Supplementary Figure 2, which additionally demonstrates Iba1 and CD58 immunoreactivity in these cell populations.

### Blood-CSF barrier (7–10 wpc)

**Figure 4:**
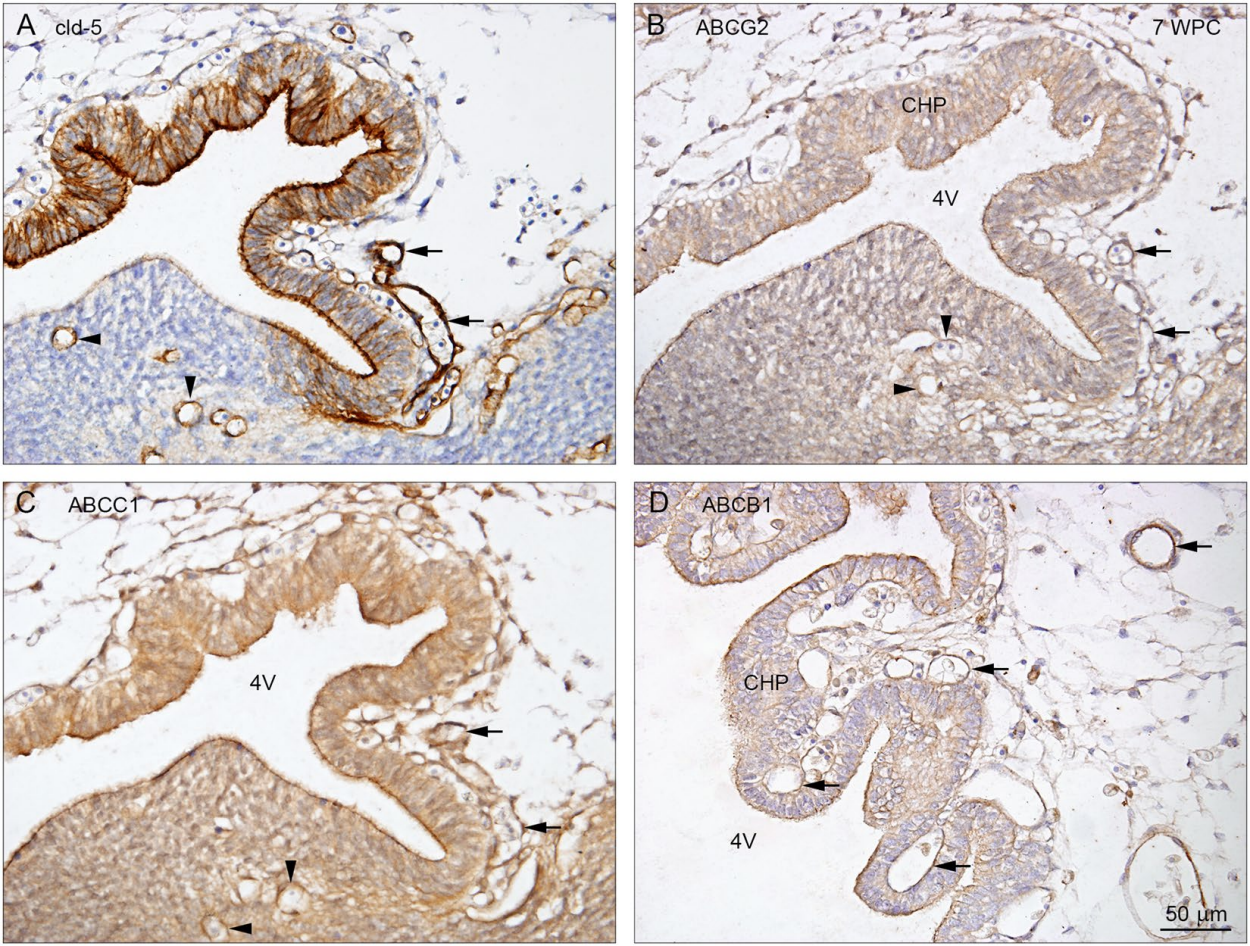
Claudin-5, ABCG2, ABCC1 and ABCB1 in the early developing 4^th^ ventricular choroid plexus at 7 wpc. Immunostaining of the emerging 4^th^ ventricle choroid plexus at 7 wpc reveals claudin-5 in apical/luminal and basolateral membranes (**A**) and ABCG2 (**B**), ABCC1 (**C)**, and ABCB1 (**D**) immunoreactivity in apical/luminal membranes of choroidal epithelial cells facing the 4^th^ ventricle (4 V) and in the fenestrated vasculature (arrows) (**A**–**D**). Note also the immunostaining of parenchymal blood vessels (arrowheads) illustrating the blood-brain barrier proper. Figures shown from 2 individual embryos. Abbreviations: CHP: choroid plexus; cld-5: claudin-5; 4 V: fourth ventricle. WPC: weeks post conception. (**A**–**D**) Same magnification. *Scale bar:* 50 μm.

**Figure 5:**
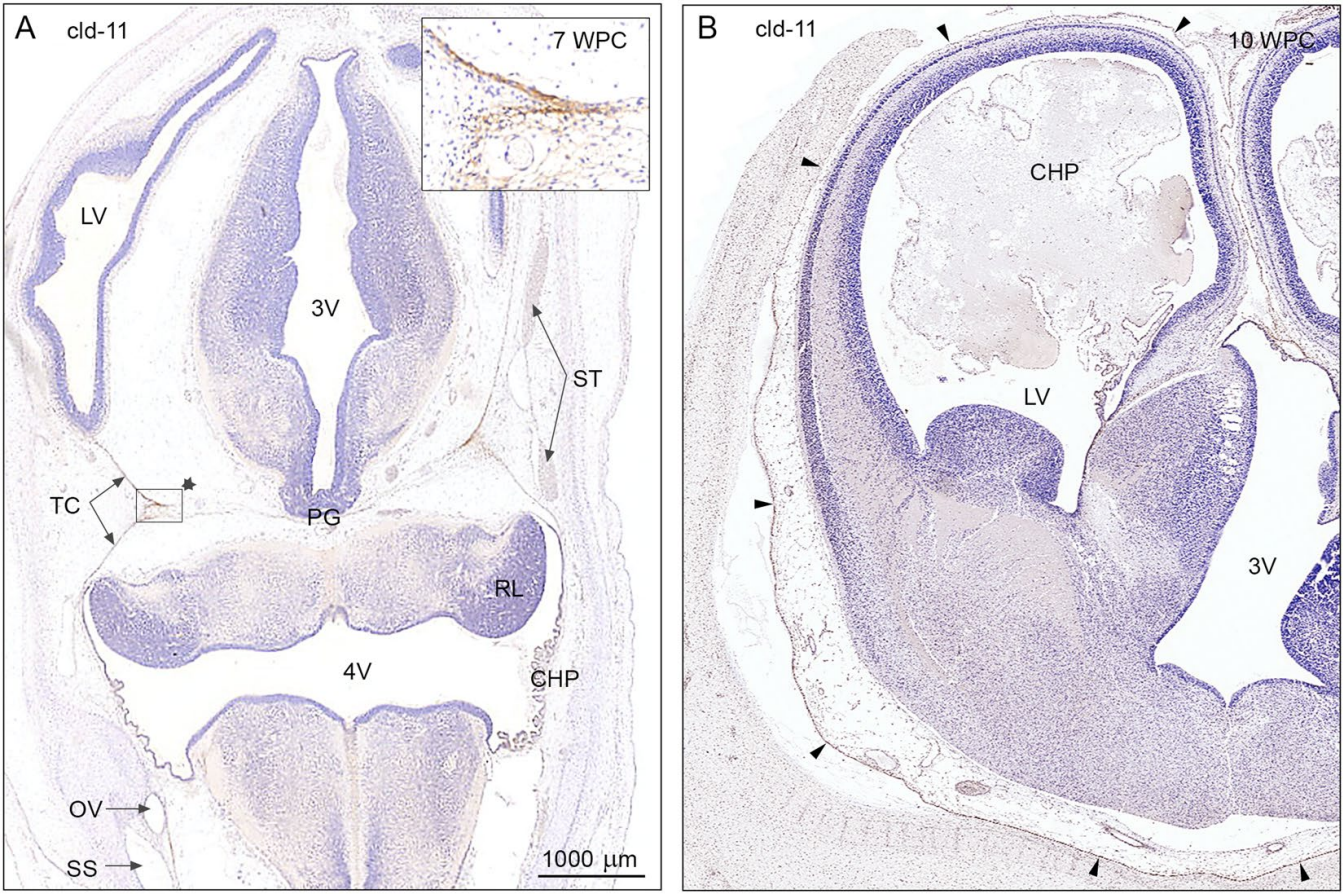
Claudin-11 immunoreactivity in a 7 wpc embryo and a 10 wpc fetus. **(A)** 7 wpc embryo: the arachnoid barrier cell layer appears initially covering the inner surface of the tentorium cerebelli (TC); it is most prominent in the medial part where the ‘blades’ of the tentorium meet (insert). Note that there is no indication of a claudin-11 positive arachnoid layer covering the forebrain (**A**). The choroid plexus (CHP) of the 4^th^ ventricle (4 V), positively immunostained for claudin-11, is starting to differentiate, and the sinuses are already present. **(B)** 10 wpc fetus: the basal forebrain shows a strongly claudin-11 immunopositive arachnoid barrier cell layer (four basolateral arrowheads) creating a well-defined subarachnoidal space in contrast to the dorso-medial forebrain where the claudin-11 immunopositive arachnoid membrane fades out along the surface (three upper lateral arrowheads). Abbreviations: CHP: choroid plexus; 4 V: fourth ventricle; LV: lateral ventricle; OV: otic vesicle; PG: pineal gland; RL: rhombic lip; 3 V: third ventricle; SS: sigmoid sinus; ST: transverse sinus; TC: tentorium cerebelli; WPC: weeks post conception; cld: claudin. (**A**,**B**) Same magnification. *Scale bar*: 1000 μm.

**Figure 6:**
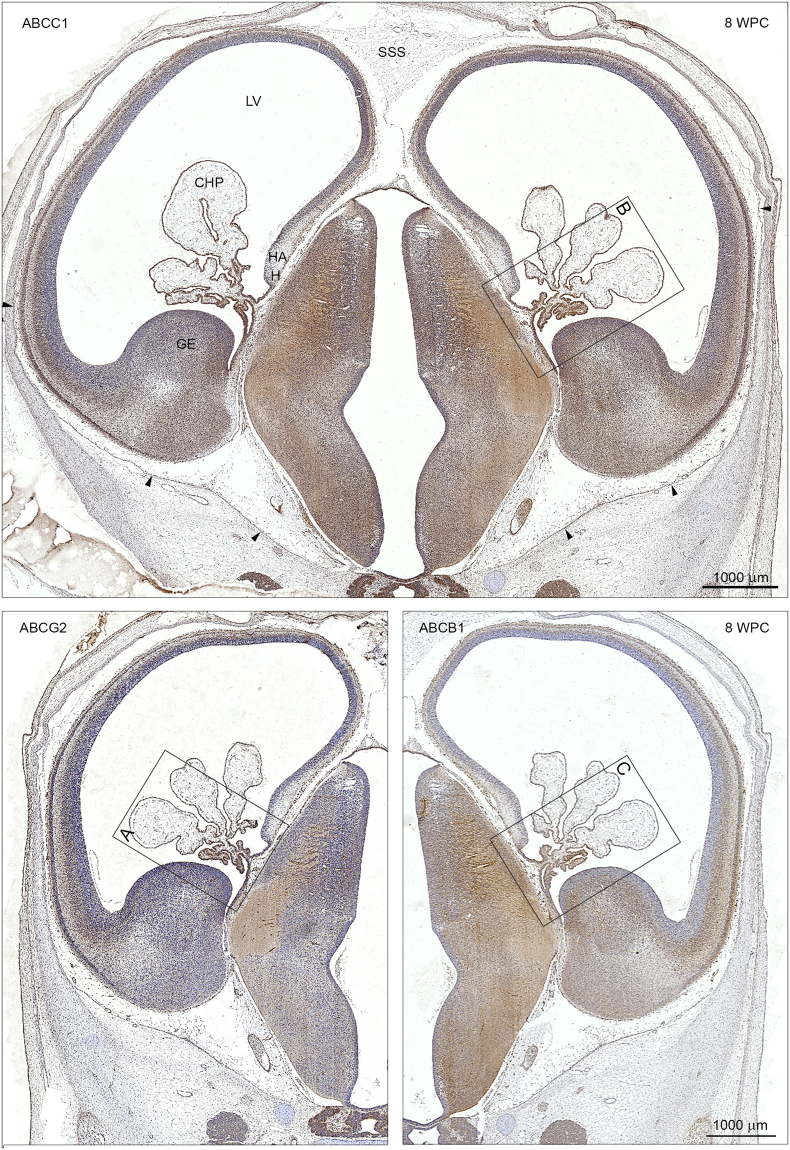
ABCC1, ABCG2 and ABCB1 immunoreactivity in coronal sections of a developing forebrain at 8 wpc. Choroid plexus epithelial cells (CHP) and brain microvasculature show a positive immunoreactivity for ABCC1, ABCG2 and ABCB1 in adjacent sections from the same fetus at this early stage of development. In order to facilitate an overall comparison the left panel of the lower figure (ABCG2) is printed as a mirror image of that of the lower right panel (ABCB1) and the boxed areas (**A**,**B**,**C**) containing the choroid plexus are shown in higher magnification in Fig. 7. The arachnoid barrier cell layer in the ventral part of the forebrain is immunoreactive (arrowheads) in contrast to the dorsal surface and the developing superior sagittal sinus (SSS). Abbreviations: CHP: choroid plexus; GE: ganglionic eminence; H: hem; HA: hippocampal anlage; LV: lateral ventricle; SSS: superior sagittal sinus. WPC: weeks post conception. *Scale bar*: 1000 μm.

Late in the 7^th^ wpc the first choroid plexus epithelium differentiates in the 4^th^ ventricle. As soon as choroidal epithelium is visible, it is immunopositive for claudins-5 (Fig. 4A) and -11 (Fig. 5A). In the next few days, lateral (Fig. 6) and finally 3^rd^ ventricular choroid plexuses appear. The blood-brain and blood-CSF barriers develop together with markers of tight junctions (claudins-5 and -11) indicating that their permeability properties are likely to have been established. In a previous study of the distribution of AFP and other plasma proteins in developing human choroid plexus based on the same material more than 30% of early plexus epithelial cells showed a positive, clearly cellular AFP immunoreactivity from basolateral to apical membranes. There was no sign of paracellular reactivity and perivascular brain areas were negative for AFP^17^.

### ABC transporters

**Figure 7:**
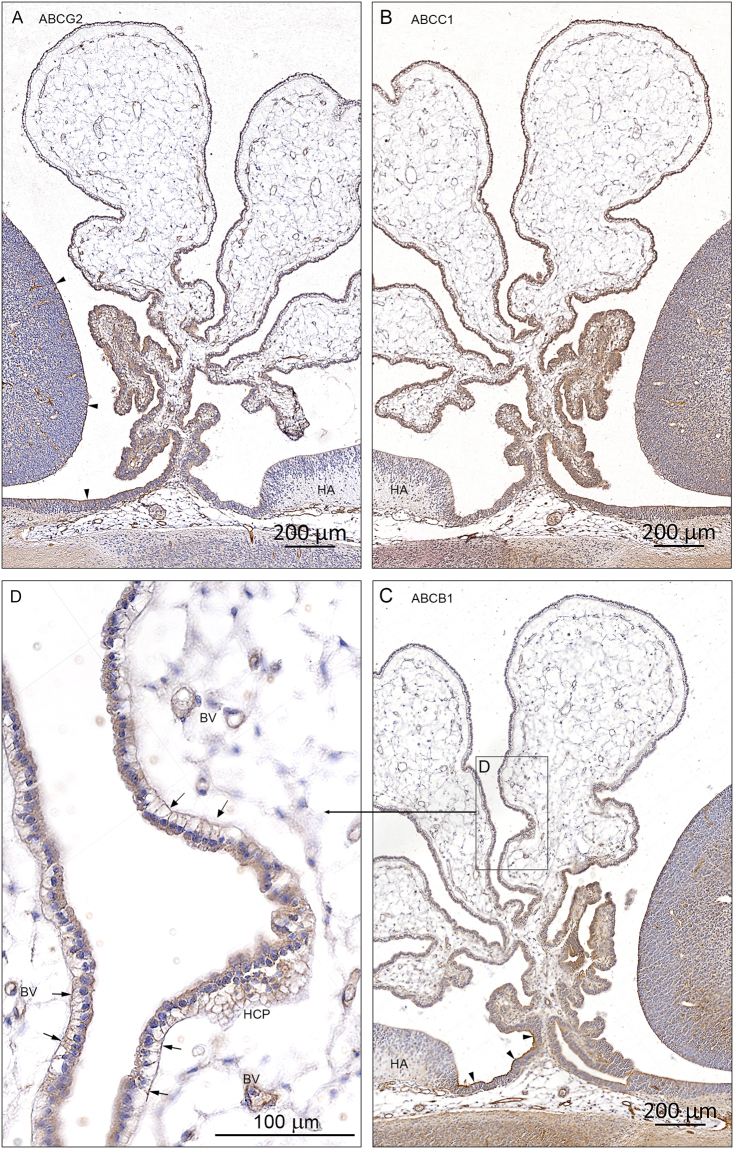
Cellular distribution of ABCG2, ABCC1 and ABCB1 immunoreactivity in early lateral choroid plexus (8 wpc). Note obvious differences in the immunostaining for ABCG2 (**A**), ABCC1 (**B**) and ABCB1 (**C**). The root of the plexus is positively stained in both cytoplasm and along apical and basolateral membranes in all efflux transporters but the leaflets of epithelial cells leading from either the ganglionic eminence or the hippocampal anlage (HA) show a different immunoreactivity. In the ABCG2 stained section **(A)** there is a marked apical membrane immunoreactivity extending from the surface of the ganglionic eminence along the ganglionic leaflet (arrowheads) to the root of the plexus whereas the leaflet connecting the root with the hippocampal anlage (HA) is unstained. In the ABCC1 stained section (**B**) both leaflets seem to be more equally reacting. In the ABCB1-immunostained section **(C)** there is very strong apical membrane reactivity (arrowheads) along the leaflet connecting the root with the hippocampal anlage (HA) in contrast to the leaflet towards the ganglionic eminence. However, closer to the ganglionic eminence the surface neuroepithelial layer becomes as immunopositive as that covering the eminence. In a higher magnification of a peripheral part of the plexus (boxed area **D**) it is obvious that the ABCB1 immunoreactivity is most pronounced along the basolateral membranes (arrows in **D**) also visualized in tangential sectioned epithelium as a honeycomb pattern (HCP). Endothelial cells of the fenestrated blood vessels (BV) are also positive. Even at the low magnification in (**A**,**B** and **C**) it is possible to recognize positive blood vessels in the ganglionic eminences and in the subarachnoid space below the root of the choroid plexus. Abbreviations: BV: blood vessel; HA: hippocampal anlage; HCP: honeycomb pattern. (**A**–**C**) Same magnification. *Scale bar*: 200 μm. (**D**) *Scale bar*: 100 μm.

In the initial stages of choroid plexus epithelium differentiation, immunoreactivity of all three ABC efflux transporters is present on the luminal/apical surface and in the fenestrated vasculature (Fig. 4B,C,D). As the plexus grows into the lateral ventricle (Fig. 6), there is change in the cellular distribution of all three transporters. In the root of the plexus there is a strong reactivity in the apical surface, however in the more distal part the immunoreactivity becomes basolateral (Fig. 7). There are also regions of the plexus, which are devoid of any immunoreactivity for the transporters.

There are obvious differences in the pattern of distribution for ABCG2 (Fig. 7A), ABCC1 (Fig. 7B) and ABCB1 (Fig. 7C) not only between the different efflux transporters *per se* but also within a given plexus stained for one of the transporters. The root of the plexus is positively stained in both cytoplasm and along apical and basolateral membranes for all three transporters but the leaflets of epithelial cells leading from either the ganglionic eminence or the hippocampal anlage show a different distribution of immunoreactivity. ABCG2 shows marked apical membrane immunoreactivity extending from the surface of the ganglionic eminence along the ganglionic leaflet to the root of the plexus, whereas the leaflet connecting the root with the hippocampal anlage is unstained (Fig. 7A). ABCC1 immunoreactivity is present in both leaflets (Fig. 7B) while ABCB1 immunostaining is very strong in the apical membrane along the leaflet connecting the root with the hippocampal anlage in contrast to the leaflet towards the ganglionic eminence. However, closer to the ganglionic eminence the surface neuroepithelial layer becomes as positive as that covering the eminence (Fig. 7C). At higher magnification of a more peripheral part of the plexus (Fig. 7D) it is obvious that the ABCB1 immunoreactivity is most pronounced along the basolateral membranes also visualized in tangential sectioned epithelium as a honeycomb pattern (HCP). Endothelial cells of the fenestrated blood vessels are also positive.

### Blood-CSF barrier (mid-gestation)

#### ABC transporters

**Figure 8:**
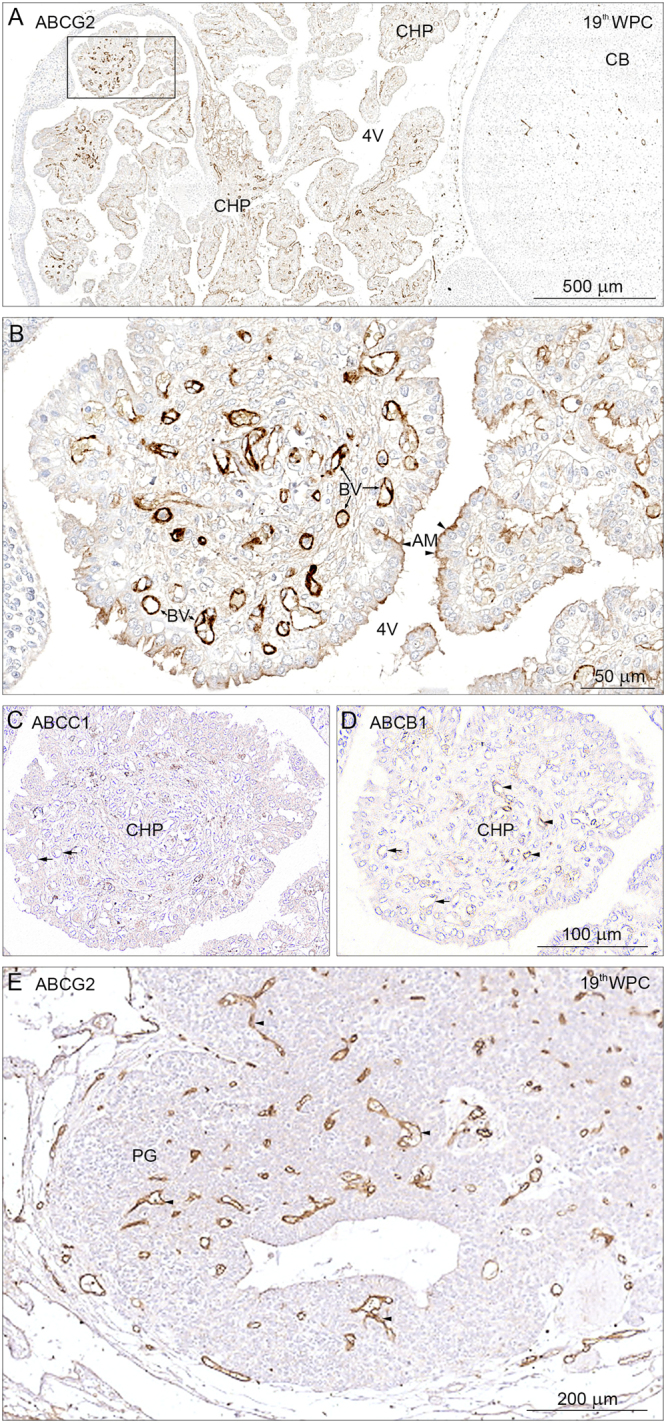
ABCG2, ABCC1 and ABCB1 in the choroid plexus, cerebellum and the pineal gland at 19^th^ wpc. Horizontal section through choroid plexus (CHP), cerebellum (CB) and pineal from a 19 wpc fetus. The microvessels on the surface of and within cerebellum (CB) are strongly immunopositive for ABCG2 **(A)** and can be easily identified even at this low magnification. In 4^th^ ventricular choroid plexus (4 V) there is marked immunoreactivity of the fenestrated endothelial cells and of patches of the apical epithelial membranes. The boxed area is shown in higher magnification in (**B**). Endothelial cells of the fenestrated blood vessels (BV) depict a very strong immunoreactivity, which seems to fill the entire cytoplasm from luminal to abluminal membranes. Patches of subregions of the plexus show a distinct luminal/apical membrane (AM) immunoreactivity indicated by arrowheads but there is no sign of basolateral membrane staining. In (**C**) slender processes of stromal macrophages are immunopositive for ABCC1 whereas there is no immunostaining of the fenestrated blood vessels (small arrows) and no immunoreactivity in the plexus epithelium. In (**D**) the vasculature of the plexus shows a differential immunostaining for ABCB1 revealing some unstained blood vessels (small arrows) whereas others show a weak signal (arrowheads). There is no immunoreactivity in epithelial cells. Like choroid plexus, most circumventricular organs are characterized by fenestrated blood vessels. The pineal gland (PG) in (**E**) exhibits ABCG2 immunopositive endothelial cells like those of the 4^th^ ventricular choroid plexus shown in (**B**). Abbreviations: AM: apical membrane; BV: blood vessel; CB: cerebellum; CHP: choroid plexus; 4 V: fourth ventricle, CHP: choroid plexus; PG: pineal gland, WPC: weeks post conception. *Scale bars*: (**A**) 500 μm, (**B**) 50 μm. (**C**,**D**) Same magnification. *Scale bar*: 100 μm; (**E**) *Scale bar*: 200 μm.

As the choroid plexus development progresses (up to mid-gestation) there is another distinct change in the distribution of the three transporters. ABCG2 immunoreactivity is strongly, but not uniformly present on the apical surface of the plexus (illustrated for 4^th^ choroid plexus in Fig. 8). The strongest immunoreactivity is demonstrated in the fenestrated blood vessels (Fig. 8B). ABCG2 is also present in blood vessels in the neighboring cerebellum (Fig. 8A). In contrast to ABCG2, immunoreactivity for both ABCC1 and ABCB1 is absent in choroid plexus epithelial cells but ABCB1 is still present in some fenestrated blood vessels (Fig. 8C,D). Circumventricular organs, part of the exchange system between the brain and the CSF, are characterized by fenestrated blood vessels similar to those of the choroid plexus stroma. They follow the pattern of choroid plexuses, i.e. they are also immunopositive for all three transporters up to 13 wpc, but at mid-gestation only ABCG2 shows strong reactivity as shown in the pineal gland (Fig. 8E).

#### Arachnoid barrier (7–10 wpc)

At this stage of brain maturation there is a clear distinction in the pattern of development between blood-brain and blood-CSF barriers and the complex arachnoid barrier. This latter interface develops over a prolonged period of weeks before there is a well-defined subarachnoid space, which includes the forebrain in contrast to blood-brain and blood-CSF barriers, which appear tight from their emergence in various brain regions and ventricles.

At 7 wpc the arachnoid barrier cell layer as shown by claudin-11 staining appears initially covering the inner surface of the tentorium cerebelli; it is most prominent in the medial part where the two ‘blades’ of the tentorium meet (insert in Fig. 5A). There is no indication of a claudin-11 positive arachnoid barrier cell layer covering the forebrain. At 10 wpc the basal forebrain shows a strongly claudin-11 positive arachnoid barrier cell layer, whereas the claudin-11 positive arachnoid membrane fades out along the dorso-medial surface (Fig. 5B) indicating that at this stage the infratentorial, fossa cranii posterior related part of the developing brain has a well-defined subarachnoidal space in contrast to the forebrain. Only when the arachnoid formation is completed in 12^th^ wpc is the entire subarachnoid space delineated and thus defined.

#### Arachnoid barrier (mid-gestation)

By mid-gestation fully developed subarachnoid interfaces are established^18^. The subarachnoid space is delineated by the arachnoid barrier cell layer towards the dura and the end feet layer towards the brain dividing it into two individual interfaces: (i) the blood-arachnoid-outer CSF interface and (ii) the outer CSF-brain interface. In addition there is a third interface within the subarachnoid space between the outer CSF and the blood in the pial microvasculature (iii) the blood-pia microvessel-outer CSF interface. This vasculature in the subarachnoid space is characterized from the earliest development by claudin-5 positive tight junctions (Fig. 1B) that confer its barrier properties, confirmed by the exclusion of AFP.

#### ABC transporters

**Figure 9:**
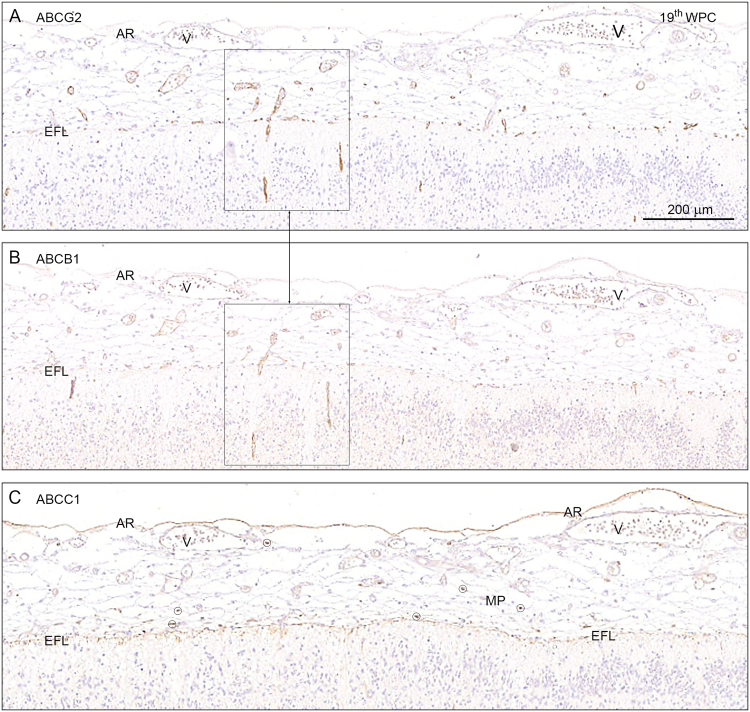
ABCG2, ABCB1 and ABCC1 in the hippocampus at 19^th^ wpc. Distribution of ABCG2, ABCB1 and ABCC1 immunoreactivity in serial frontal sections through the hippocampus of a 19^th^ wpc fetus. The subarachnoid space delineated by the arachnoid barrier cell layer (AR) towards the dura and the end feet layer (EFL) towards the brain still contains many ABCG2 and ABCB1 immunostained microvessels as shown in (**A** and **B**) but virtually no immunostaining of the vasculature for ABCC1, which seems to show immunoreactivity only of the numerous macrophages (MP), five of which are shown encircled, and of the arachnoid barrier cell layer (AR) and the end feet layer (EFL). Elongating radial vessels which extend from the subarachnoidal space into the marginal zone are immunopositive for both ABCG2 and ABCB1 as demonstrated in the two boxed areas in (**A** and **B**). Endothelium of the veins (V) shows no immunoreactivity. Abbreviations: AR: arachnoid barrier cell layer; EFL: end feet layer; MP: macrophage; V: vein, WPC: weeks post conception. (**A**–**C**) Same magnification. *Scale bar*: 200 μm.

Many ABCB1 and ABCG2 positively immunostained microvessels are visible within the subarachnoid space, SAS (Fig. 9A,B) but virtually no labeling of the vasculature for ABCC1 could be detected. ABCC1 is however present in numerous meningeal macrophages within the SAS at the end feet layer and the arachnoid barrier cell layer (Fig. 9C). Intensity of the immunostaining for ABCC1 appears to be declining after 15 wpc in the microvessels but remains at similar levels in endothelium of larger arterioles and small arteries. In contrast immunoreactivity of both ABCG2 and ABCB1 remains constant but is never detected on endothelium of small arteries. Elongating radial vessels, which extend from the subarachnoidal space into the marginal zone, are positive for both ABCG2 and ABCB1 but not for ABCC1.

#### Inner CSF-brain interface (from 5 wpc – mid-gestation)

The first fluid that is present in the brain ventricles is called eCSF^12^ and is derived from the amniotic fluid as it gets trapped following the closure of the neural tube. Secretion of the CSF begins following the appearance of the choroid plexuses^19, 20^. This now creates the inner CSF-brain interface. During development this interface changes its morphology and functional properties as has been described extensively for several species before^13, 21–23^. At early stages of sheep brain development, for example, this interface is characterized by the presence of specific and specialized junctional complexes called strap junctions; these junctional complexes disappear as the brain matures and are replaced by gap junctions^22^. Strap junctions have also been identified in early fetal human brain^24^.

#### ABC transporters

The immunoreactivity for ABC transporters at the stage before choroid plexuses appear has been described earlier in this paper and illustrated in Figs 2 and 4. Once choroid plexuses are present within the brain ventricles, the inner CSF-brain interface is immunopositive for ABCG2 and ABCB1 at the ganglionic eminence but less so for ABCC1 (Fig. 6). The immunopositivity for ABCB1 covers the entire ganglionic eminence down to the antihem from where the inner dorsolateral neocortical wall and the medial wall including the hippocampal anlage (HA) are devoid of signals. As described earlier in the result section, immunostaining of the inner CSF-brain interface shows marked regional differences clearly related to the area-specific differentiation of neuroepithelium to ependyma occurring over a broad range around mid-gestation. The outer CSF-brain interface differentiates from an endfeet layer to a glia limitans after mid-gestation with concomitant changes in ABC transporters.

## Discussion

**Figure 10:**
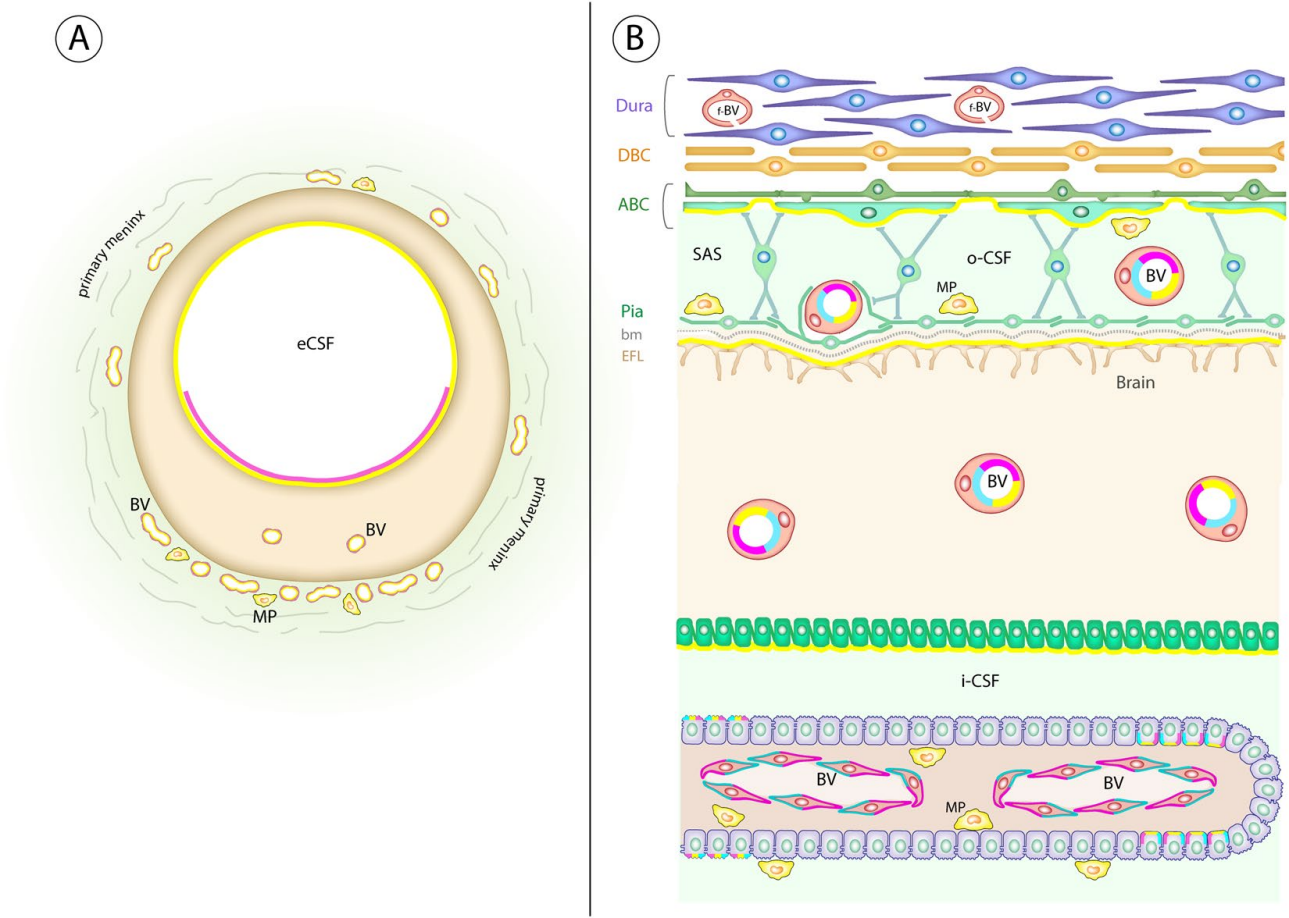
Schematic diagram of ABCG2, ABCC1 and ABCB1 distribution in an early human embryonic brain anlage and in a 13 wpc fetal brain. Diagram summarizes the data showing the spatial change in the distribution of ABCG2 (cyan), ABCC1 (yellow) and ABCB1 (magenta) in the early human embryonic brain anlage (**A**) and a 13 wpc fetal brain (**B**). (**A**): at 5 wpc ABCB1 (cyan) is not present in the brain anlage, ABCG2 (magenta) immunoreaction is seen in parts of the ventral eCSF-brain interface, whereas ABCC1 (yellow) is detected over the whole of this interface as well as in macrophages located in the primary meninx. Both ABCG2 and ABCC1 stain blood vessels of the perineural vascular plexus in the primary meninx as well as the first incoming brain parenchymal vessels. (**B**): at 13 wpc all three ABC transporters are at their maximum density of immunostaining compared to any other time during the first half of gestation. After this period there is a progressive decline in the intensity of ABCB1 and ABCC1 immunoreactivity. All three ABC transporters (cyan, magenta and yellow) are detected in brain parenchymal blood vessels, blood vessels in the subarachnoid space and subpopulations of choroid plexus epithelial cells at 13 wpc. In addition, ABCB1 and ABCG2 immunostaining is present in fenestrated blood vessels of the choroid plexuses (cyan and magenta) and ABCC1 (yellow) is present at several interfaces: the outer subarachnoid space, the outer surface of the brain and the inner CSF-brain interface; it is also present in macrophages in the choroid plexus stroma and subarachnoid space. See also Table 2. Abbreviations: ABC: arachnoid barrier cells; bm: basement membrane; BV: blood vessel; DBC: Dural border cells; EFL: end feet layer; f-BV: fenestrated blood vessel; i-CSF: inner cerebrospinal fluid; MP: macrophage; oCSF: outer cerebrospinal fluid; tj: tight junction.

Limiting entry of potential neurotoxins to the brain is an essential function of brain barrier mechanisms, both during development and in the adult brain. This study of developing brain barriers and efflux mechanisms in human embryos and fetuses in the first half of gestation is the first in a series of two. Part two will describe second part of human gestation. Data presented so far provide evidence for two important aspects of early human brain development:

(i) Distribution of immunostaining for the human fetal plasma protein AFP shows clearly that this protein, and presumably others in plasma, is excluded from the brain as soon as the neural tube closes and even before blood-brain barrier itself is present. This taken together with the finding that claudin-5 and/or -11 are already present in every interface as each appears suggests that barriers in the human embryonic and fetal brain are “tight” as described in other animal species^1, 5^.
(ii) The sequential appearance of three major ABC efflux transporters in the various brain barriers and interfaces as they appear in development suggests developmental adaptations at specific stages of brain maturation. In addition their cellular distribution also changes as the development progresses and their presence seems to be strongest at around 13 wpc, which is schematically indicated and described in Fig. 10 and Table 1 as a general summary of the results so far. The potential clinical importance of these findings is discussed below.

**Table 1:** Summary of results as illustrated in Fig. 10.

Interfaces Present		ABCB1 (PGP)		ABCG2 (BCRP)		ABCC1 (MRP1)	
**Age: 5 wpc**	**Primary Meninx**						
	blood vessels	−		++		+	
	macrophages	−		−		++	
	**Blood-brain barrier proper**						
	blood vessels	−		++		+	
	**Inner eCSF-brain interface**						
	neuroepithelium dorsal	−		−		+	
	neuroepithelium ventral	−		+		+	
	**Arachnoid barrier**						
**Age: 13 wpc**	arachnoid barrier cell layer	−		−		++	
	subarachnoid macrophages	−		−		+++	
	pia microvessels	+++		+++		++	
	pial end feet layer	−		−		++	
	**Blood-brain barrier proper**						
	blood vessels	+++		+++		++	
	**Blood-CSF barrier**						
	blood vessels	+++		+++		−	
	choroid plexus epithelium*	+++	−	+++	−	+++	−
	choroid plexus macrophages	−		−		+++	
	**Inner CSF-brain interface**						
	neuroepithelium/ependyma	−		−		++	

*Staining present on a sub-population of epithelial cells. For details see legend to Fig. 10.

In the early stages of vascularization of the brain a loose vascular ectomesenchyme rostral to the mesencephalon derived from the neural crest continues caudally along the brain stem and spinal cord as a vascular mesenchyme originating from the paraxial mesoderm. This layer of loose mesenchymal tissue, which surrounds the neural tube and the developing early brain anlage has been termed the primary meninx (see refs 13, 18). Prior to the development of the dura and the leptomeninges (arachnoid, subarachnoid space and pia) an extensive microvascular network of interdigitating small arterioles, capillaries and venules termed the perineural vascular plexus (PNVP) is established by vasculogenesis at the surface of the neural tissue. Following differentiation of the leptomeninges from 7 to 10 wpc (see below) the PNVP will form the vasculature in the subarachnoid space.

Vascularization of the human brain has been described in a number of studies^25–27^. Following the progressive proliferation of blood vessels over the surface of the brain, primordial vessels grow into the brain parenchyma from the primary meninx of the ventral surface of the developing brain. It has been suggested that this process begins in the 6^th^ - 7^th^ wpc^28, 29^. It is clear from the present study that the embryonic brain is protected from circulation-derived molecules such as AFP, even before the process of intrinsic angiogenesis begins. The first vessels penetrating into the brain parenchyma of the human embryo have characteristic brain barrier properties: they exclude AFP and are immunopositive for claudin-5.

Hemodynamics has been shown to have a major impact on arteriovenous specification and patterning^28^. Nevertheless evidence from animal studies indicates that endothelial cells in the developing brain are regulated by compartment-specific homeobox transcription factors and give rise to pial vessels and periventricular vessels, which most likely form venous sinuses and arterial networks, respectively^30, 31^. Considering the differential expression of ABC transporters in brain vasculature in the present study, it is intriguing whether at least some of the ABC transporters relate to only the arterial or venous network.

There appear to be only very few reports describing ABC transporters in the human fetal brain with diverging results. Only the blood-brain barrier and blood-CSF barrier have been evaluated in detail. ABC efflux transporters that appear to be of particular importance in brain barriers are ABCB1 and ABCG2 at the blood-brain barrier and ABCC1 at blood-CSF barrier^10^. ABCB1 is believed to act as a “flippase” transporting lipid-soluble substrates from the inner leaflet of the plasma membrane to the extracellular fluid^32^, as opposed to ABCC1 and ABCG2 that bind their substrates in the cell cytoplasm^10^. Considering the role of ABC transporters in neuroprotection and pharmacoresistance^33^ mapping their presence and spatiotemporal appearance in developing human brain will contribute to important knowledge of their functions and interactions.

ABCB1 is expressed in a variety of human tissues^34, 35^. A wide range of endogenous substrates and therapeutic drugs are substrates for this transporter^34–36^. In the developing human brain the presence of ABCB1 in brain capillaries at 28 weeks gestation but not earlier was reported^37^. Weak immunopositive staining for P-glycoprotein (ABCB1) in cerebral endothelial cells as early as 30 mm crown rump length (8 wpc), using the JSB-1 monoclonal antibody has been identified^38^. This was only present in the midbrain and brainstem regions. Later in gestation the endothelial immunostaining became progressively more pronounced and could be visualised in blood vessels of all major parts of the brain. The presence of P-glycoprotein has been confirmed in cerebral vascular endothelial cells at 12, 18 and 22 weeks gestation (corresponding to 10, 16, and 20 wpc) using four different monoclonal antibodies including JSB-1^39^. Immunocytochemically detectable P-glycoprotein, BCRP (ABCG2) and MRP-1 (ABCC1) in mid- to late-gestation human fetuses (22–42 weeks gestation) compared to adult brain was also reported^40^. P-glycoprotein was only detected in brainstem and, “other regions of the hindbrain, and thalamus”, with no staining of forebrain vessels between 22 and 26 weeks gestation but an increase in immunostaining intensity in older fetuses^40^. However, the material was from autopsies conducted 18–20 hours after death on live born neonates that died from a variety of pathologies, consequently much of the material is poorly fixed. Furthermore since several neurological diseases modulate the activity, location and expression of ABC transporters^41^, this may interfere with results. These considerations may account for some of the differences in immunostaining pattern between various studies^38, 40^. In contrast to the above findings a separate study reported that ABCB1 immunostaining was predominantly on the surface of neural stem/progenitor cells from 9 weeks gestation and was only detected in brain blood vessels in the adult^42^. In the present study ABCB1 immunostaining was found in placenta at 5 wpc, on brain endothelial cells about one week later and was strongest at the blood-brain barrier around 13 wpc. Prior results of ABCB1 in choroid plexus suggest low levels, but subcellular and cellular localization remains unclear and data are mostly derived from animal studies^7, 10^. We found ABCB1 in fenestrated vasculature in choroid plexus and a spatiotemporal gradient of immunostaining at the apical and basolateral surface of the choroid plexus epithelial cells, (which could explain some of the conflicting results in literature). At mid-gestation ABCB1 was no longer present in choroid plexus epithelium, but immunoreactivity was still found in fenestrated vessels in choroid plexus and microvessels within the subarachnoid space. Our study therefore supports the presence of ABCB1 in the early blood-brain barrier, in vessels in the subarachnoid space and differential appearance in choroid plexus, although we did not see increasing staining after 13 wpc.

ABCC1 is considered to be a marker of the blood-CSF barrier^33, 40^, but evidence characterizing ABCC1 in human development is sparse. ABCC1 was identified in choroid plexus epithelial cells, but not in cerebral endothelial cells at all ages from 22 weeks gestation and older^40^. ABCC1 was found in both the blood-CSF barrier and blood-brain barrier in rats and human adult samples, although in a much lower level at the blood-brain barrier and with differences in reported activity^7, 43^. We found ABCC1 immunostaining in developing brain from 5 wpc; initially in the perineural vascular plexus in the ventral part of the upper spinal cord, the hindbrain and the midbrain, parenchymal blood vessels, the whole eCSF-brain interface, placenta, Hofbauer cells and tissue macrophages in the primary meninx. As was the case for ABCB1 and ABCG2, ABCC1 was present in choroid plexus epithelial cells and fenestrated vasculature from the earliest development of choroid plexus, although the spatiotemporal distribution was different, and at mid-gestation ABCC1 was no longer present in the choroid plexus. ABCC1 immunoreactivity was seen in both the CSF-brain barrier, and at the level of the arachnoid barrier cell layer and end feet layer in the arachnoid barrier. It also stained meningeal macrophages, but was sparse in the vasculature in SAS, in contrast to ABCB1 and ABCG2 immunoreactivity.

ABCG2 is thought to work synergistically with ABCB1 limiting transport through the blood-brain barrier^33^. In previous studies ABCG2 was identified in human fetal brain microvessels at all ages and brain regions examined from 22 weeks gestation and older, but not in choroid plexus^40^. Its immunostaining did not appear to vary with maturation. ABCG2 was shown to be associated with choroidal vessels but not choroidal epithelium during development in transgenic mice^44^. In the present studies, at 5 wpc we found ABCG2 immunoreactivity in perineural vascular plexus and parenchymal blood vessels, placenta and eCSF-brain interface as for ABCC1, but with a less prominent and consistent staining in the eCSF-brain interface. In contrast to the two other transporters, the presence of ABCG2 was widespread in both choroid plexus epithelial cells and fenestrated vasculature of the plexuses at all ages examined and in microvessels in the subarachnoid space.

It is widely believed that the developing fetus and in particular its central nervous system is vulnerable to damage from maternally administered drugs^45^. Drugs used to treat neurological disorders (e.g. epilepsy or mental illness) enter CNS because they are not substrates for protective efflux mechanisms at brain barriers or their functional capacity has been exceeded. In the developing brain it is possible that the functional capacity may be less than in the adult, thus allowing entry of a drug that would be excluded in the adult. Prescriptions for pregnant women and neonates are generally based on clinical experience and reports of adverse effects^46^. Notwithstanding placental protection, drugs have the potential to enter developing brain. The detailed description of when three of the main ABC efflux transporters appear in early brain development and particularly the finding that ABCG2 is present from the earliest stage of central nervous system development, just after closure of the neural tube, suggests that some degree of brain protection may already be present even in the embryo. This study provides an initial stage of the process aiming to understand efflux mechanisms operating in barriers in the developing human brain. This understanding is required in order to develop a rational basis for the safer prescription of drugs in pregnant women.

This study is a comprehensive investigation of ABC transporters in very early developing human brain. Assessing functional capacities of efflux transporters and efficacy of barrier mechanisms in the embryonic and fetal human brain is crucial for determining risk of fetal damage with maternal drug use^45^. The present results, demonstrating the morphological integrity of brain interfaces and presence of ABC transporters in these barriers even at the earliest stages in development, indicate that embryonic and early fetal brain may be better protected than expected with external (placenta) and internal (brain barriers) defense systems. Nevertheless spatiotemporal diversity in ABC transporter appearance, possible effects of genetic variability^34^ on ABC transporter function, modulation by other endogenous and exogenous compounds^41^ and interplay with other defense systems (such as placenta) paint a complex picture and complicate medical practice and risk assessment.

ABC transporter-mediated efflux protects the brain against toxic assaults, but unfortunately also prevents relevant therapeutic drugs from reaching the brain. ABC transporters are enhanced in several neurological disorders^41^, which further limits drug penetration to the brain. Considering the broad substrate specificity and overlap of ABC transporters, inhibiting ABC transporters as a mean of increased penetration of specific drugs seems difficult^10^.

Further functional studies and a description of ABC transporters in second part of gestation are necessary to map the exact function and interaction of efflux transporters in the developing brain, which may help assessing risk of drug use for pregnant women and reveal new methods of increasing drug transport to the brain.

## Materials and Methods

### Tissue samples

Brains from ten human embryos (6–31 mm crown-rump length (CRL)) and 11 fetuses (38–200 mm CRL) corresponding to 5^th^-21^st^ weeks post conception (wpc) were examined. The embryos and the fetuses were obtained from legal abortions, as previously described^47^. Informed consent was obtained from all contributing women following oral and written information, in accordance with the Helsinki declaration II, and approved by the Research Ethics Committee of the Capital Region (KF–V.100.1735/90). Full medical history of the women is also known. Post-operational treatment of tissue consisted of immediate dissection of the samples into blocks and subsequent fixation for 12–24 hours at 4 °C in either 10% neutral buffered formalin, 4% Formol-Calcium, Lillie’s or Bouin’s fixatives. This procedure kept the time from delivery to fixation at a minimum, normally less than 2 hours, in order to retain optimal tissue quality. Most of the embryos were collected in relation to studies of smoking and alcohol in pregnancy and for schizophrenia in families. The numbers of embryos/fetuses available for the study were: 5–6 wpc, 5; 7 wpc, 5; 8–11 wpc, 4; 12–15 wpc 6; 19–20 wpc, 2. For immunohistochemistry, 2–10 μm thick serial sections were cut in transverse, sagittal or horizontal planes, and placed on silanized glass slides.

### Immunohistochemistry

Sections were deparaffinized and rehydrated in xylene following standard protocols. When necessary antigen retrival was performed (see Table 2) by 10 min boiling in citrate buffer, pH 6 or TEG pH 9. Endogenous peroxidase was quenched using a 0.5% solution of hydrogen peroxide in TBS for 15 min. Following rinses with TRIS buffered saline (TBS, 5 mM Tris-HCl, 146 mM NaCl, pH 7.6), non-specific binding was inhibited by incubation for 30 min with 10% normal goat serum at room temperature. The sections were incubated overnight at 4 °C with primary antibodies (Table 2) diluted in 10% normal goat serum and washed with TBS. For bright field light microscopy analysis, the REAL EnVision Detection System (Peroxidase/DAB + rabbit/mouse, code K5007, DakoCytomation, Glostrup, Denmark) was used for detecting mouse and rabbit primary antibodies. The sections were washed with TBS, followed by incubation for 10 min with the DAB + solution. Positive staining was recognized as a brown color. The sections were counterstained with Mayers hematoxylin and dehydrated in graded alcohols and coverslipped with Pertex mounting medium.

**Table 2:** List of primary antibodies.

Primary antibodies	Host IgG	Dilution	Retrieval	Producer	Code number
ABCB1/PGP	Mouse IgG1	1:15–1:30	TEG	Abcam	ab3366
ABCC1/MRP1	Mouse IgG2a	1:30–1:50	M6	Abcam	ab24102
ABCG2/BCRP	Mouse IgG2a	1:15–1:20	M6	Abcam	ab3380
AFP	Rabbit IgGs	1:6000	—	Dako	A008
CD 34	Mouse IgG1	1:25	—	Dako	M7165
Claudin-5	Rabbit IgG	1:200	M6	Abcam	ab15106
Claudin-11/OSP	Rabbit IgG	1:800	—	Abcam	ab53041

Details of the primary antibodies including dilutions and suppliers are listed in Table 2. Control sections were incubated with mouse IgG1, IgG2a or irrelevant rabbit antibodies, as well as subjected to omission of primary or secondary antibodies. These were always blank. As positive controls we used placental tissue as the syncytiotrophoblastic cell layer is strongly immunopositive for ABCG2, ABCB1 and ABCC1, and the prominent placental stromal macrophages (Hofbauer cells) are positive for ABCC1.

### Data Availability

The data that support the findings of this study are available from [Dr K Møllgård] but restrictions apply to the availability of these data, which were used under license for the current study, and so are not publicly available. Data are however available from the authors upon reasonable request and with permission of [Dr K Møllgård].

## Electronic supplementary material

Supplementary Figures

## References

[1] Saunders NR, Liddelow SA, Dziegielewska KM (2012). Barrier mechanisms in the developing brain. Front Pharmacol.

[2] Brightman MW, Reese TS (1969). Junctions between intimately apposed cell membranes in the vertebrate brain. J Cell Biol.

[3] Saunders NR, Daneman R, Dziegielewska KM, Liddelow SA (2013). Transporters of the blood-brain and blood-CSF interfaces in development and in the adult. Mol Aspects Med.

[4] Liddelow SA (2013). Mechanisms that determine the internal environment of the developing brain: a transcriptomic, functional and ultrastructural approach. PLoS ONE.

[5] Saunders NR (2014). The rights and wrongs of blood-brain barrier permeability studies: a walk through 100 years of history. Front Neurosci.

[6] Kratzer I (2013). Developmental changes in the transcriptome of the rat choroid plexus in relation to neuroprotection. Fluids Barriers CNS.

[7] Strazielle N, Ghersi-Egea J-F (2015). Efflux transporters in blood-brain interfaces of the developing brain. Front Neurosci.

[8] Grandjean P, Landrigan PJ (2006). Developmental neurotoxicity of industrial chemicals. Lancet.

[9] Vasiliou V, Vasiliou K, Nebert DW (2009). Human ATP-binding cassette (ABC) transporter family. Hum Genomics.

[10] Saunders, N. R., Habgood, M. D., Møllgård, K. & Dziegielewska, K. M. The biological significance of brain barrier mechanisms: help or hindrance in drug delivery to the central nervous system? *F1000Res* **5**(F1000 Faculty Rev**)** 313 (2016). 10.12688/f1000research.7378.1 PMC4786902 26998242

[11] Ek CJ (2010). Efflux mechanisms at the developing brain barriers: ABC-transporters in the fetal and postnatal rat. Toxicol Lett.

[12] Bueno D, Parvas M, Hermelo I, Garcia-Fernàndez J (2014). Embryonic blood-cerebrospinal fluid barrier formation and function. Front Neurosci.

[13] O’Rahilly R, Müller F (1986). The meninges in human development. J Neuropathol Exp Neurol.

[14] Gitlin D (1975). Normal biology of alpha-fetoprotein. Ann N Y Acad Sci.

[15] Gitlin, D. & Biasucci, A. Development of gamma G, gamma A, gamma M, beta IC-beta IA, C 1 esterase inhibitor, ceruloplasmin, transferrin, hemopexin, haptoglobin, fibrinogen, plasminogen, alpha 1-antitrypsin, orosomucoid, beta-lipoprotein, alpha 2-macroglobulin, and prealbumin in the human conceptus. *J Clin Invest* 1433–1446 (1969). 10.1172/JCI106109 PMC322370 5796355

[16] Møllgård K, Jacobsen M (1984). Immunohistochemical identification of some plasma proteins in human embryonic and fetal forebrain with particular reference to the development of the neocortex. Brain Res.

[17] Jacobsen M, Jacobsen GK, Clausen PP, Saunders NR, Møllgård K (1982). Intracellular plasma proteins in human fetal choroid plexus during development. II. The distribution of prealbumin, albumin, alpha-fetoprotein, transferrin, IgG, IgA, IgM, and alpha 1-antitrypsin. Brain Res.

[18] Brøchner CB, Holst CB, Møllgård K (2015). Outer brain barriers in rat and human development. Front Neurosci.

[19] Johanson CE, Reed DJ, Woodbury DM (1974). Active transport of sodium and potassium by the choroid plexus of the rat. J Physiol (Lond).

[20] Johansson PA, Dziegielewska KM, Liddelow SA, Saunders NR (2008). The blood-CSF barrier explained: when development is not immaturity. Bioessays.

[21] Fossan G (1985). CSF-brain permeability in the immature sheep fetus: a CSF-brain barrier. Brain Res.

[22] Møllgård K, Balslev Y, Lauritzen B, Saunders NR (1987). Cell junctions and membrane specializations in the ventricular zone (germinal matrix) of the developing sheep brain: a CSF-brain barrier. J Neurocytol.

[23] Whish S (2015). The inner CSF–brain barrier: developmentally controlled access to the brain via intercellular junctions. Front Neurosci.

[24] Møllgård K, Saunders NR (1986). The development of the human blood-brain and blood-CSF barriers. Neuropathol Appl Neurobiol.

[25] Mall FP (1905). On the Development of the blood-vessels of the brain in the human embryo. Am J Anat.

[26] Padget DH (1948). The development of the cranial arteries in the human embryo. Contr Embryol.

[27] Allsopp G, Gamble HJ (1979). Light and electron microscopic observations on the development of the blood vascular system of the human brain. J Anat.

[28] Raybaud C (2010). Normal and abnormal embryology and development of the intracranial vascular system. Neurosurg Clin N Am.

[29] Duckett S (1971). The establishment of internal vascularization in the human telencephalon. Acta Anat (Basel).

[30] Vasudevan A, Bhide PG (2008). Angiogenesis in the embryonic CNS: a new twist on an old tale. Cell Adh Migr.

[31] Vasudevan A, Long JE, Crandall JE, Rubenstein JLR, Bhide PG (2008). Compartment-specific transcription factors orchestrate angiogenesis gradients in the embryonic brain. Nat Neurosci.

[32] Higgins CF, Gottesman MM (1992). Is the multidrug transporter a flippase?. Trends Biochem Sci.

[33] Qosa H, Miller DS, Pasinelli P, Trotti D (2015). Regulation of ABC efflux transporters at blood-brain barrier in health and neurological disorders. Brain Res.

[34] Lam J, Koren G (2014). P-glycoprotein in the developing human brain: a review of the effects of ontogeny on the safety of opioids in neonates. Therap Drug Monit.

[35] Cordon-Cardo C (1990). Expression of the multidrug resistance gene product (P-glycoprotein) in human normal and tumor tissues. J Histochem Cytochem.

[36] Mahringer A, Fricker G (2016). ABC transporters at the blood-brain barrier. Expert Opin Drug Metab Toxicol.

[37] van Kalken CK (1992). Multidrug resistance gene (P-glycoprotein) expression in the human fetus. Am J Pathol.

[38] Schumacher U, Møllgård K (1997). The multidrug-resistance P-glycoprotein (Pgp, MDR1) is an early marker of blood-brain barrier development in the microvessels of the developing human brain. Histochem Cell Biol.

[39] Virgintino D (2008). Fetal blood-brain barrier P-glycoprotein contributes to brain protection during human development. J Neuropathol Exp Neurol.

[40] Daood M, Tsai C, Ahdab-Barmada M, Watchko JF (2008). ABC transporter (P-gp/ABCB1, MRP1/ABCC1, BCRP/ABCG2) expression in the developing human CNS. Neuropediatrics.

[41] Miller DS (2015). Regulation of ABC transporters at the blood-brain barrier. Clin Pharmacol Ther.

[42] Yamamoto A (2009). ABCB1 is predominantly expressed in human fetal neural stem/progenitor cells at an early development stage. J Neurosci Res.

[43] Gazzin S (2008). Differential expression of the multidrug resistance-related proteins ABCb1 and ABCc1 between blood-brain interfaces. J Comp Neurol.

[44] Orford M (2009). Generation of an ABCG2(GFPn-puro) transgenic line–a tool to study ABCG2 expression in mice. Biochem Biophys Res Commun.

[45] Goasdoué K, Miller SM, Colditz PB, Björkman ST (2016). Review: The blood-brain barrier; protecting the developing fetal brain. Placenta.

[46] Briggs, G. G. & Freeman, R. K. *Drugs in Pregnancy and Lactation*. 10^th^ edition (Wolters Kluwer, 2015).

[47] Bjørnbak C, Brøchner CB, Larsen LA, Johansen JS, Møllgård K (2014). Brain Barriers and a Subpopulation of Astroglial Progenitors of Developing Human Forebrain Are Immunostained for the Glycoprotein YKL-40. J Histochem Cytochem.

