# Supplementary material for: Brain barriers and functional interfaces with sequential appearance of ABC efflux transporters during human development
Source: Sci Rep. 2017 Sep 14;7:11603. doi: 10.1038/s41598-017-11596-0 (PMC5599687; doi:10.1038/s41598-017-11596-0)
Supplement: Supplementary file 1 — Supplementary Figures [file 41598_2017_11596_MOESM1_ESM.pdf]

Brain barriers and functional interfaces with sequential appearance of ABC efflux transporters during human development.

Kjeld Møllgård, Katarzyna M Dziegielewska, Camilla B Holst, Mark D Habgood, Norman R Saunders

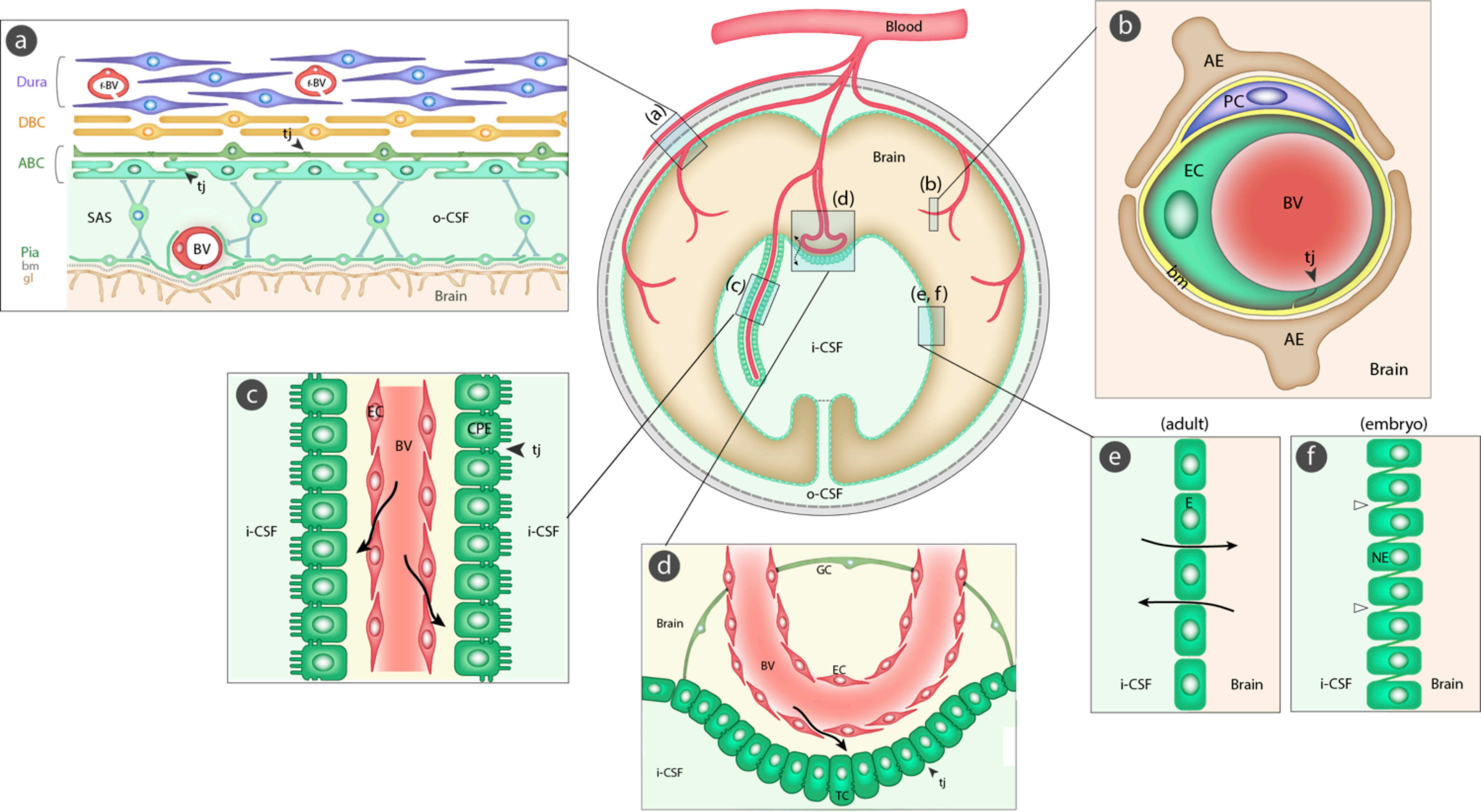

a
Dura
f-BV
DBC
tj
ABC
tj
SAS
o-CSF
Pia
bm
gl
BV
Brain
Blood
(a)
Brain
(b)
(d)
(c)
(e, f)
i-CSF
o-CSF
b
AE
PC
EC
BV
tj
bm
AE
Brain
c
EC
BV
CPE
tj
i-CSF
i-CSF
d
GC
Brain
BV
EC
i-CSF
TC
tj
(adult)
(embryo)
e
E
i-CSF
Brain
f
NE
i-CSF
Brain

Supplementary Figure 1

Schematic diagram (centre left) of the five main brain barrier interfaces (a-e) in the adult and an additional one in the embryo (f). The barrier-forming cellular layers at each interface are in green.

This figure has been adapted from Saunders et al. (2016)[10]. (a) *The arachnoid barrier* is structurally the most complex of all the brain barriers. Barrier forming cells are the arachnoid barrier cells (ABC) with tight junctions (tj, arrowheads) between adjacent cells forming a barrier between the outer CSF (oCSF) in the subarachnoid space (SAS) and more superficial dural layers (dural border cells, DBC and the dura mater). Blood vessels (BV) in the SAS have tight junctions with similar barrier characteristics as cerebral blood vessels without surrounding pericytes and astrocytic end-feet. Blood vessels within the dura mater are fenestrated (f-BV). (b) *The blood-brain barrier proper* is situated at the level of cerebral blood vessels (BV), with tight junctions (tj, arrowhead) between endothelial cells (EC). (c) *The blood-CSF barrier* is situated in the choroid plexus within each brain ventricle, with apical tight junctions (tj, arrowhead) between epithelial cells (CPE). Although endothelial cells in the blood vessels (BV) are connected by tight junctions (not shown), they are fenestrated and do not form a barrier (arrows); apical microvilli increase exchange surface of epithelial cells to the internal CSF (iCSF). (d) *The circumventricular organ (CVO) barrier* (including median eminence, pineal gland, area postrema, subfornical organ) is formed by tanycytes (TC), the specialized ependymal cells of these brain areas, connected by tight junctions between their apices (arrowhead). (e) *The adult CSF-brain barrier*. Apart from areas where there are specialised tanycytes, ependymal cells are linked by gap junctions that do not restrict exchange of even large molecules, such as proteins, between CSF and the interstitial space of the brain (solid arrows). (f) *The embryonic CSF-brain barrier.* In early brain development, strap junctions (open arrowheads) are present between adjacent neuroepithelial cells (NE); these form a barrier restricting movement of larger molecules such as proteins, but not smaller molecules.

Abbreviations: ABC: arachnoid barrier cells; AE: end feet from astroglial cells; bm: basement membrane; BV: blood vessel; DBC: dural border cells; E: ependymal cell; EC: endothelial cell; f-BV: fenestrated blood vessel; gl: glia limitans; i-CSF: inner cerebrospinal fluid; NE: neuroepithelial cell; oCSF: outer cerebrospinal fluid; PC: pericyte; SAS: subarachnoid space; tj: tight junction.

Figure adapted from[48].

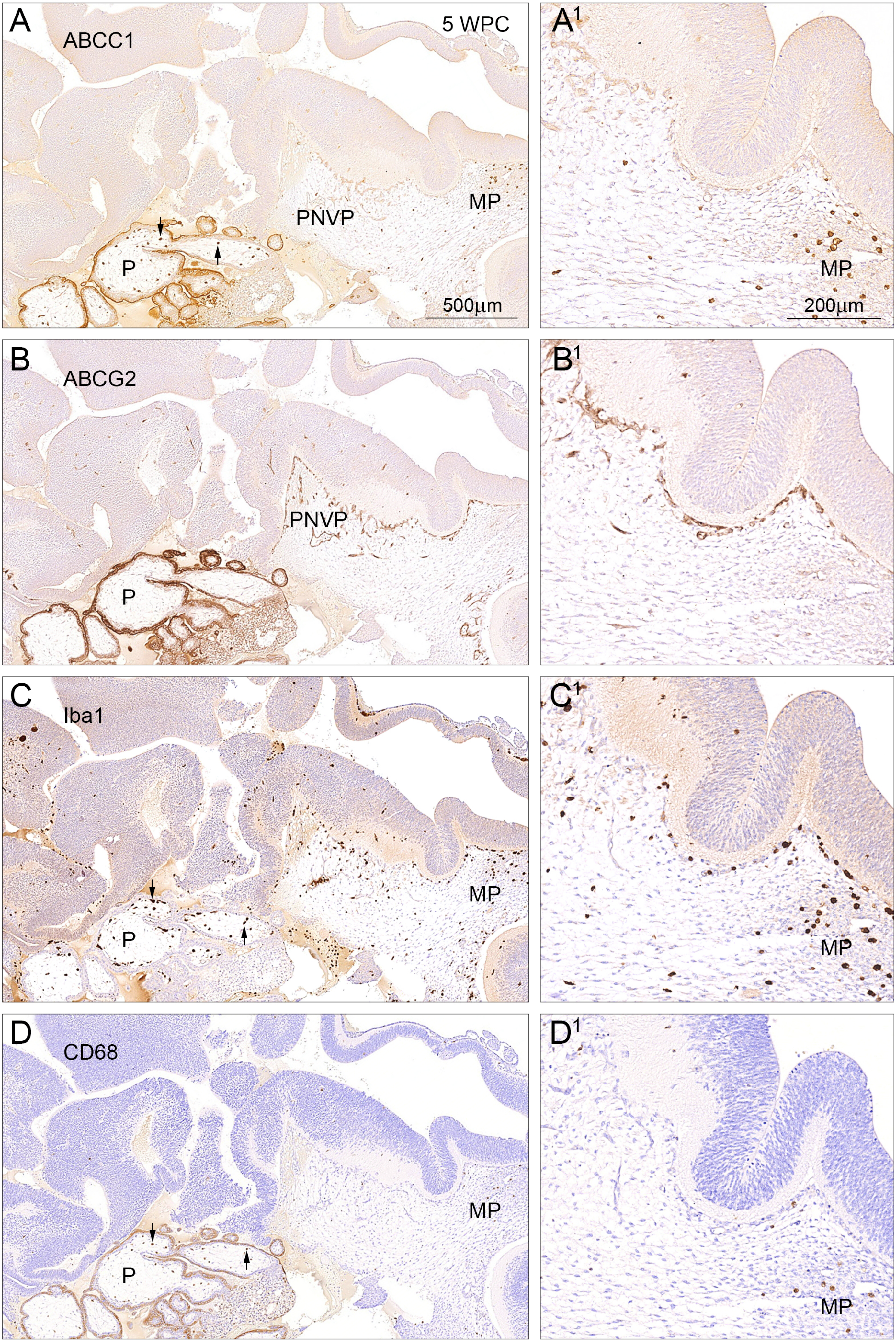

A
ABCC1
5 WPC
PNVP
MP
P
500µm
A1
MP
200µm
B
ABCG2
PNVP
P
B1
C
Iba1
MP
P
C1
MP
D
CD68
MP
P
D1
MP

Supplementary Figure 2

Distribution of ABCC1, ABCG2, Iba1 and CD68 immunoreactivity in 4 consecutive sagittal sections of a 5 wpc human embryonic brain anlage.

Immunostaining for ABCC1 (**A**) and ABCG2 (**B**) depicts the same dense perineural vascular plexus (PNVP) in the ventral part of the mid- and hindbrain as shown in Figure 2. Immunoreactivity of the specific markers for macrophages (Iba1 in **C**, and CD68 in **D**) are shown in two consecutive sections. The many macrophages (MP) immunopositive for ABCC1 (**A**) shown in higher magnification in $\mathbf{A^1}$, are clearly positive for Iba1 (**C** and $\mathbf{C^1}$) and a subgroup is positive for CD68 (**D** and $\mathbf{D^1}$). Note however, that this cell group is not seen following staining for ABCG2 (**B** and $\mathbf{B^1}$). The placental tissue (P) included in the sections (**A-D**) contains prominent stromal macrophages (Hofbauer cells) also positive for ABCC1, Iba1 and CD68 (arrows), but not for ABCG2 (**B).**

Abbreviations: MP: macrophages; P: placental tissue; PNVP: perineural vascular plexus.

A - D: Same magnification. *Scale bar*: 500 µm. $\mathbf{A}^1$ - $\mathbf{D}^1$: Same magnification. *Scale bar*: 200 µm.

Staining for Iba1 and CD68 was carried out without heat induced antigen retrieval using anti-Iba1 rabbit IgG (1:250) from DAKO (019-19741) and anti-CD68 mouse IgG1 (1:400) from DAKO (M0814).
